# Four women whose pioneering contributions to science have been largely overlooked

**DOI:** 10.7554/eLife.110644

**Published:** 2026-02-11

**Authors:** Lisa M Thomann, Julie Batut

**Affiliations:** 1 Centre de Recherche en Biologie Cellulaire de Montpellier (CRBM), Université de Montpellier, CNRS Montpellier France; 2 Association Femmes & Sciences Paris France; 3 Unité de Biologie Moléculaire, Cellulaire et du Développement (MCD, UMR 5077), Centre de Biologie Intégrative (CBI, FR 3743), Université de Toulouse, CNRS Toulouse France

**Keywords:** Equity, Diversity and Inclusion, women in science, history of science, Spemann-Mangold organizer, Down syndrome, None

## Abstract

Ethel Browne Harvey and Hilde Pröscholdt Mangold did pioneering research in embryology, Ida Henrietta Hyde helped develop the first microelectrodes for the stimulation of single cells, and Marthe Gautier had a vital role in discovering that Down syndrome is caused by an extra copy of chromosome 21. So why are their names so little known by the scientific community at large?

## Introduction

The history of science is rarely as simple as 'what happened in the past'. Instead, it is often a version of events that focuses on the successes of great names, mostly men, and tends to overlook other contributions, in particular contributions made by women. Examples include Nettie Stevens and Rosalind Franklin in the biological sciences, and Lise Meitner and Chien-Shiung Wu in the physical sciences. To help redress this imbalance, we highlight here the pioneering contributions made by four women to the biological sciences. We hope that this article will serve as a reminder that women have always been part of science, despite what the history books say and what lists of Nobel prize-winners might suggest.

Why are so few women mentioned in histories of science? An obvious factor is that women were outnumbered by men in science for many years, but this cannot be the whole reason. Are women underrepresented in histories of science because of a lack of talent and/or ambition on their part? Or is it because of a systematic pattern of undercounting the number of women in science and minimising their contribution to science – a phenomenon christened the ‘Matilda effect’ by Margaret W Rossiter, the American historian of science, in Rossiter, 1993.

Understanding the gender differences in scientific output has been the focus of extensive research in recent decades, and a number of factors that impact the scientific productivity of women have been explored. These include: women being underrepresent in more senior positions in laboratories; hostile work environments and supervision; and differences in work-life balance (Ross et al., 2022; Rossiter, 1993; Bostwick and Weinberg, 2022; Valian, 2018; Feldman, 2023; Ceci and Williams, 2011; Tomaskovic-Devey, 2005; Derrick et al., 2022). In particular, a study published in 2022 reported that at least some of the gender gap in scientific output is due to a systematic lack of recognition of women’s contribution, with their work being under-appreciated or simply ignored (Ross et al., 2022).

Making science more inclusive, for everyone, is one of the main challenges we have to tackle as scientists today. We have to empower scientists to bring women who contributed to science throughout history out of the shadows. We need to start filling the gaps in various histories of science to highlight the contribution of women to what science is today.

In this article, we will tell the stories of four women scientists who made important contributions to the field of developmental biology – Ethel Browne Harvey, Hilde Pröscholdt Mangold, Ida Henrietta Hyde and Marthe Gautier – but were pushed into the shadows until recently. With the exception of Hilde – who died tragically young – they all travelled back and forth across the Atlantic to learn new techniques and to pursue important scientific questions.

We will conclude this section with the words of Margaret Rossiter. “The sexist nature of much of the women’s systematic under-recognition should be acknowledged, noted and even highlighted in the sociology of knowledge or science”, she wrote in 1993. Rossiter argued that this under-recognition should be called the ‘Matilda effect’, similar to how the ‘Matthew effect’ is used to describe how the “rich get richer” in science. The Matthew effect takes its name from a passage in the Gospel of Matthew, and Rossiter felt that the second half of this passage – about the poor getting poorer – applied to women in science. She chose the name Matilda effect to honour Matilda J Gage, an American writer and activist who had “experienced and articulated this phenomenon” in the late nineteenth century (Rossiter, 1993).

Earlier in her article, Rossiter writes: “Not only have those unrecognised in their own time generally remained so, but others that were well-known in their day have since been obliterated from history, either by laziness or inertia, or by historians with definite axes to grind.” Unless we take a more proactive approach in bringing forward women’s contribution into science, this pattern will continue.

## Ethel Browne Harvey (1885–1965)

**Figure 1.**
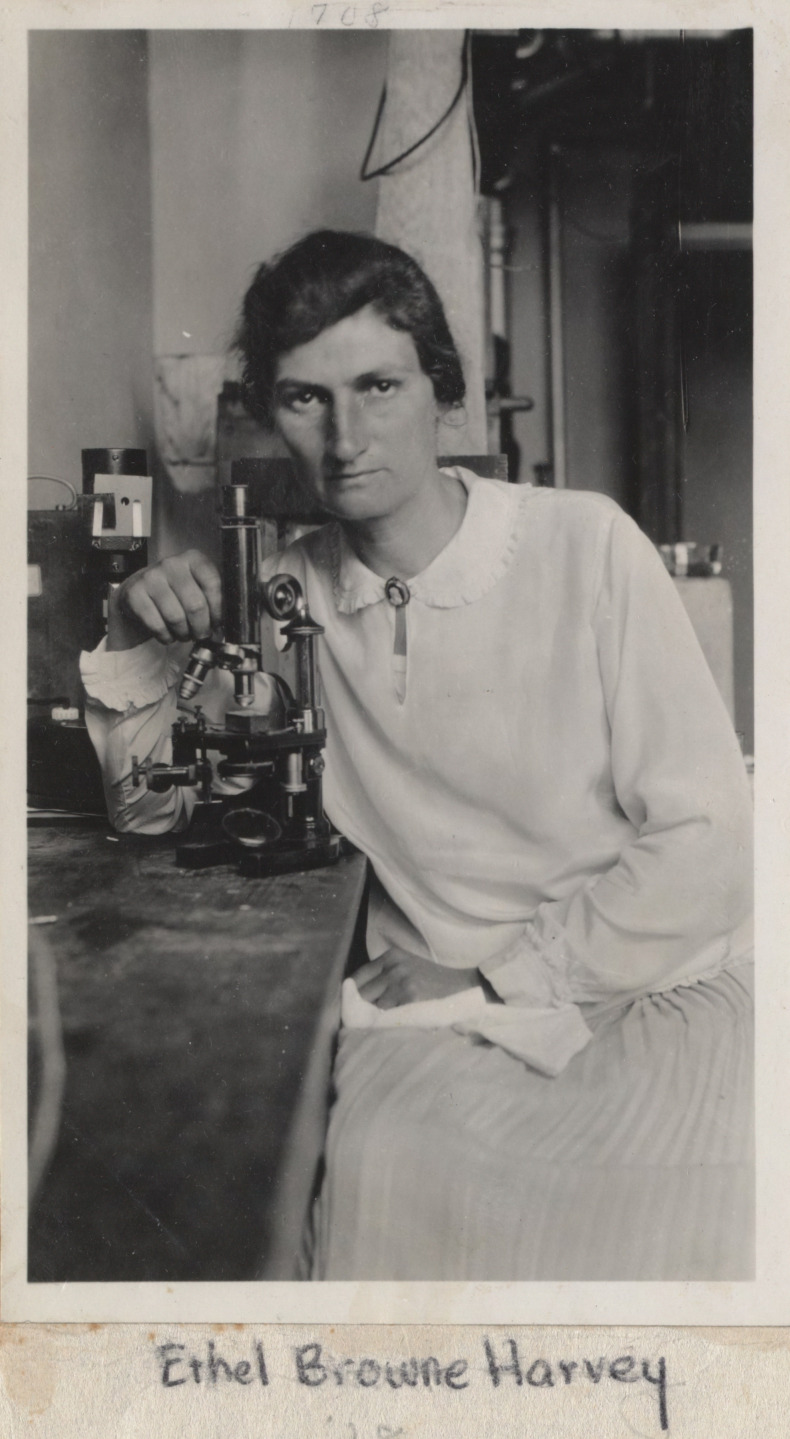
Ethel Browne Harvey. Born in Baltimore on 14 December 1885, Ethel Browne Harvey was an embryologist who specialised in cell division. She obtained her PhD from Columbia University in 1913, and subsequently had positions at Princeton and Cornell, the Marine Biological Laboratory at Woods Hole, and the Anton Dohrn Zoological Station in Naples. She discovered what is now known as the Spemann-Mangold organiser 15 years before Hans Spemann and Hilde Pröscholdt Mangold published their work on this topic, but she was overlooked when Spemann was awarded a Nobel Prize in 1935.

Ethel Browne Harvey (born Ethel Nicholson Browne; Figure 1) is mostly known today for her research in embryology, including the first demonstration that a form of asexual reproduction known as merogony is able to occur in cells that do not have a nucleus (Harvey, 1936; Harvey, 1938). Educated and trained at Columbia University, Ethel conducted her PhD under the supervision of Edmund Beecher Wilson, best known for his contribution to chromosome theory and genetics (Dröscher, 2002). She was also mentored by Thomas Hunt Morgan, winner of the Nobel Prize for Physiology or Medicine in 1933, and widely considered to be the father of molecular genetics.

Photograph courtesy of the Marine Biological Laboratory (MBL), CC-BY 4.0.

Ethel received her PhD from Columbia in 1913 for her work on the cell biology of spermatocytes in *Noctonecta*, an aquatic insect (Browne, 1910; Browne, 1913). However, few people know that her remarkable career started four years earlier when – working on a side project with Morgan – she published a paper demonstrating for the first time the induction by a transplant of a secondary axis of polarity in the transplanted host (Browne, 1909). Indeed, while working on *Hydra*, a small freshwater hydrozoan, Ethel discovered that if she grafted hyposomal cells from the mouthpiece of a hydra to a certain site on the body of another hydra, a new hydra would develop at the point of the graft.

An important question was: does this new hydra develop from the grafted cells, following induction by the host tissue, or does the new hydra develop from the host, following induction by the grafted cells? In a series of elegant experiments, Browne took advantage of the existence of hydras with different pigmentation to answer this question. She grafted hyposomal cells from a non-pigmented hydra to the body of another hydra that belonged to the same species but had green pigmentation. She observed the development of a new green hydra at the site of the transplant, and stated that “the ectoderm and endoderm cells that have been body wall cells are therefore changed over into cells composing tentacles and hypostome”.

Indeed, she proved through multiple experiments that only the hyposomal tissue, analogous to the lip of the blastopore, was able to induce the formation of a whole individual along with a secondary body axis (Barresi and Gilbert, 2020). Ethel demonstrated conclusively that the host tissue was organised by the transplanted mouth cells, thus providing the first evidence for the existence of an organiser. However, her discovery was not given the recognition it deserved until Howard Lenhoff highlighted its importance to the field of developmental biology in Lenhoff, 1991.

Ethel’s work was the basis for subsequent work on the Spemann-Mangold organiser (Lenhoff, 1991; Holstein, 2024) by Hilde Pröscholdt Mangold (see below) in the laboratory of Hans Spemann at the University of Freiburg. Spemann went on to receive the Nobel Prize for Physiology or Medicine in 1935 "for his discovery of the organizer effect in embryonic development". It is our view that Ethel Browne Harvey should have at least shared the prize with Spemann, and that she should be much better known for her contributions to developmental biology than she is today. (Hilde Mangold could not have shared the prize with Spemann as she died in a tragic accident when she was just 26.)

## Hilde Mangold (1898–1924)

**Figure 2.**
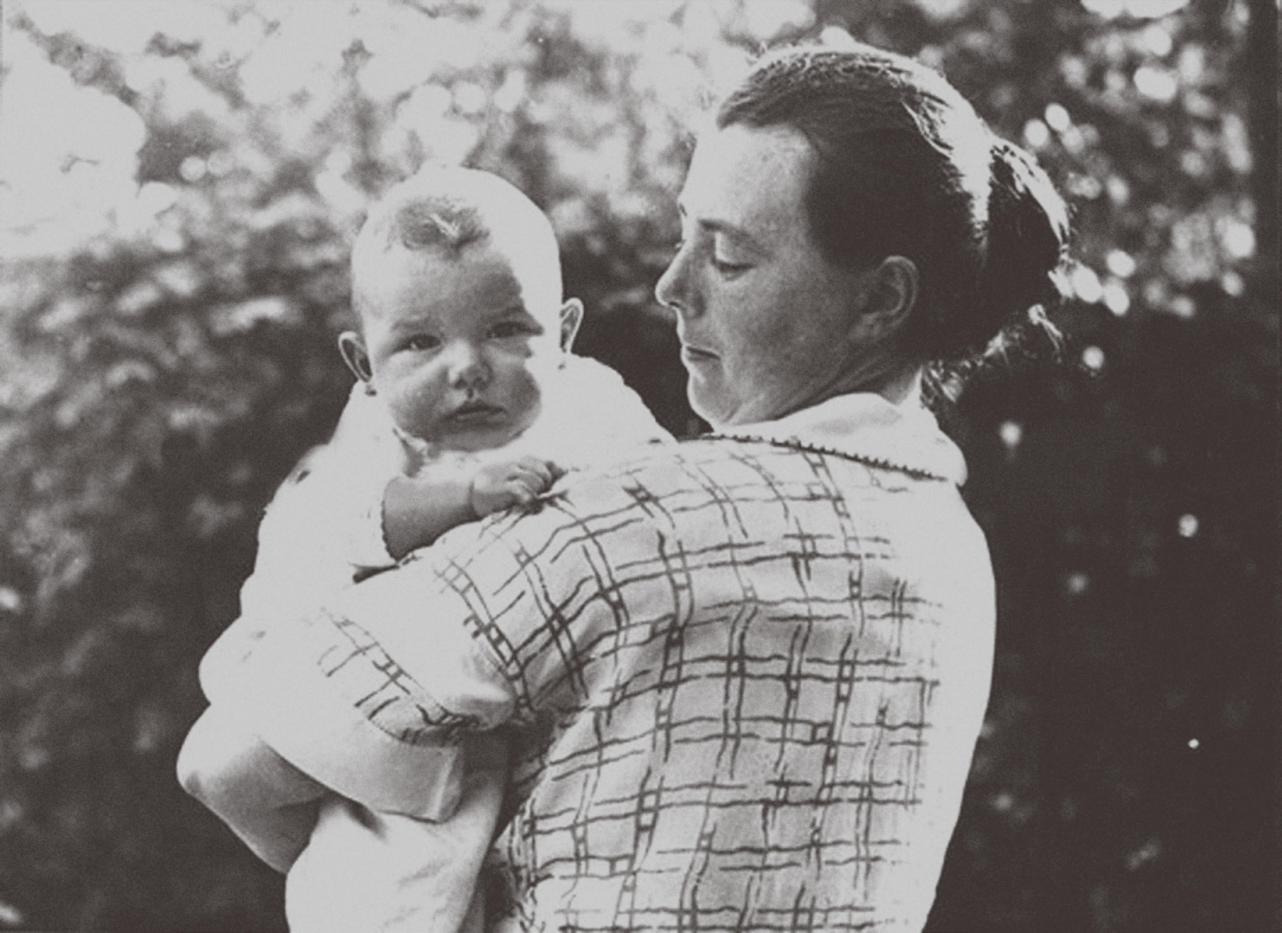
Hilde Pröscholdt Mangold. Born in Gotha, Germany on 20 October 1898, Hilde Pröscholdt Mangold was an embryologist specialising in embryonic inductions. She obtained received her PhD from the University of Freiburg in 1923 for her work on the specification of cell identity during gastrulation. Her supervisor, Hans Spemann, subsequently received the Nobel Prize for this work. Her life and career were cut short when she died in a tragic accident in 1924. She was only 26 years old.

Hilde Mangold (born Hilde Pröscholdt; Figure 2) is known today for her work as a PhD student with Hans Spemann on the organiser concept. For over two years, Hilde worked tirelessly on this project, transplanting over 250 embryos and meticulously recording the results. Only six survived the process and they were presented in her thesis, and also in a research article based on her thesis (Spemann and Mangold, 1924). Published in 1924, this article describes the transplantation of the dorsal blastopore lip of one newt gastrula embryo onto the ventral side of a host embryo at the same stage (Hamburger, 1984). It is still one of very few PhD projects to lead directly to a Nobel Prize. Unusually, Hans Spemann insisted on being listed as first author on the article based on Hilde’s PhD thesis, something he had not done with any of his male PhD students (Waelsch, 1992). Minimising the contributions of women seemed to be a normal occurrence in Spemann’s lab (Waelsch, 1992; Spemann, 1935).

Hilde died shortly after the publication of her work, and Spemann went on to receive the Nobel Prize in 1935. Hilde was only mentioned twice in Spemann’s Nobel lecture, whereas her husband Otto, who also worked with Spemann, was mentioned seven times (Spemann, 1935). Moreover, the lecture did not mention that the work being acknowledged by the prize was based on Hilde’s PhD project (Davidson and Peter, 2015).

In the years that followed Spemann was recognized for his brilliance and contribution to science, while Hilde was slowly forgotten, with many researchers referring to the Spemann-Mangold organiser as the Spemann organiser, even recently (De Robertis, 2006; Kumar et al., 2021). If it wasn’t for Hilde’s friend and co-worker, Viktor Hamburger, publishing a memoir about his time in Spemann’s lab and his friendship with Hilde, she might have been lost in time (Hamburger, 1984). Hilde was described by colleagues and friends as an unusually gifted and persevering young scientist with a penetrating and reflective intellect.

Hilde’s experiments showed a strong similarity to those of Ethel Browne Harvey. She grafted cells from the lip of the blastopore from a newt embryo at the gastrula stage onto the flank of a gastrula of an unpigmented embryo, which resulted in the induction of a secondary body axis mostly made of the unpigmented host tissue (Cousin, 2019; Driever et al., 2024). Overall, this early work by Ethel and Hilde was the first characterisation of the processes of specification and determination of cell fate, and it conclusively demonstrated the plasticity of embryonic cell fate. They also inspired later scientists to explore how cells are directed toward certain paths by cues received from their neighbours and, more generally, from their micro-environment.

## Ida Henrietta Hyde (1857–1945)

**Figure 3.**
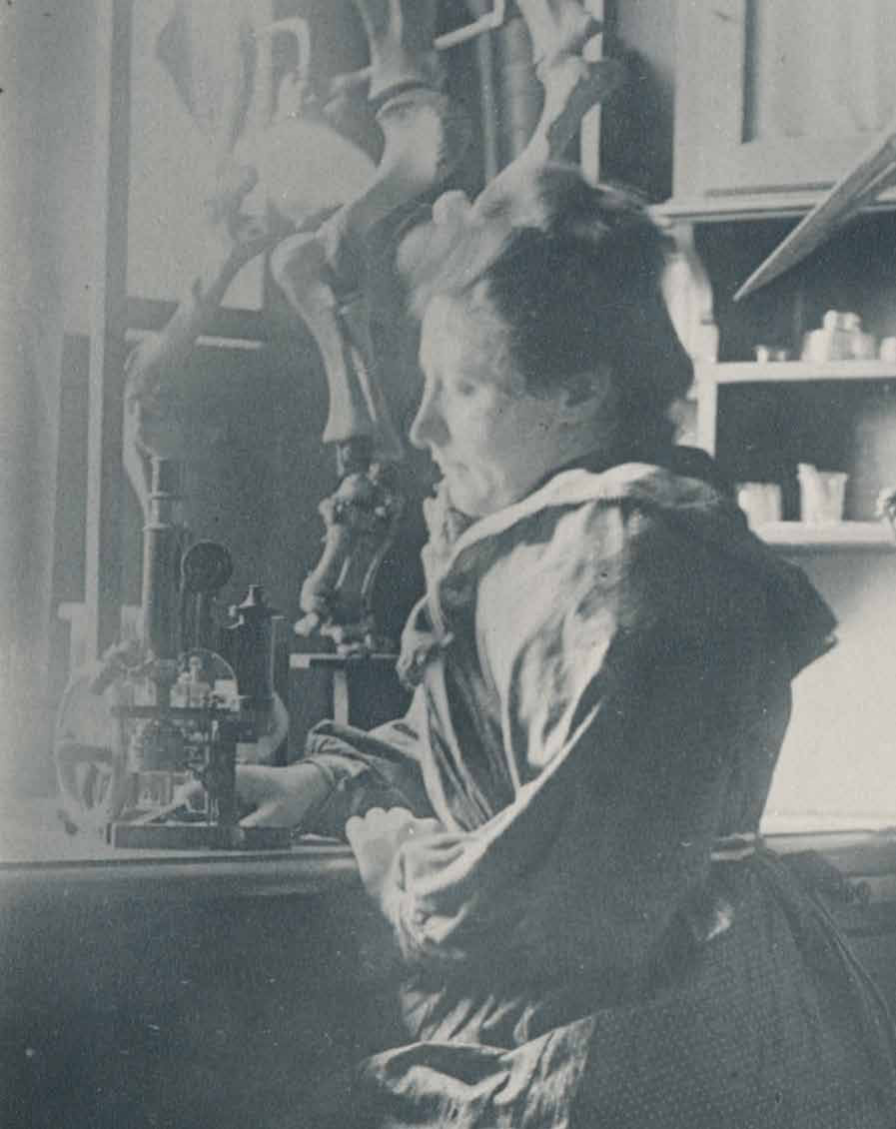
Ida Henrietta Hyde. Born in Davenport, Iowa, on 8 September 1857, Ida Henrietta Hyde specialised in neurophysiology and cellular electrophysiology. She received her PhD from the University of Heidelberg, and held positions at the University of Kansas, the Anton Dohrn Zoological Station in Naples, Harvard Medical School, and the University of Bern. She was also a tireless advocate for women in science, founding fellowships and associations to support female researchers throughout her career.

Ida Henrietta Hyde (Figure 3) was a pioneering physiologist, who is best known today for developing one of the first intracellular microelectrodes that could stimulate and make measurements on individual cells. Renowned for her ingenuity and perseverance, she also conducted foundational research on the nervous and circulatory systems of both vertebrates and invertebrates.

Unable to afford university studies, she began her career as an elementary school teacher in Chicago to save up money for her degree. She went on to be educated and trained at Cornell, Bryn Mawr and the Marine Biological Laboratory in Woods Hole.

In 1893, supported by a fellowship from the American Association of University Women, she joined the research group of Alexander Goette at the University of Strasbourg, but women were not allowed to do PhDs in Strasbourg at the time, so she transferred to the University of Heidelberg in Germany. She was allowed to attend lectures in all departments except one – for physiology she had to work independently using lecture notes taken by the Professor of Physiology’s assistants (Hyde, 1938). Still, in 1896, she became the first woman to receive a PhD in physiology from Heidelberg (Mazón, 2003).

Ida also became the first woman to do research at Harvard Medical School, when she joined the group of William Townsend Porter later in 1896. In 1898, she moved to the University of Kansas, where she went on to found the Department of Physiology and set up the Naples Table Association for Promoting Scientific Research by Women (Hyde, 1938).

## Marthe Gautier (1925–2022)

**Figure 4.**
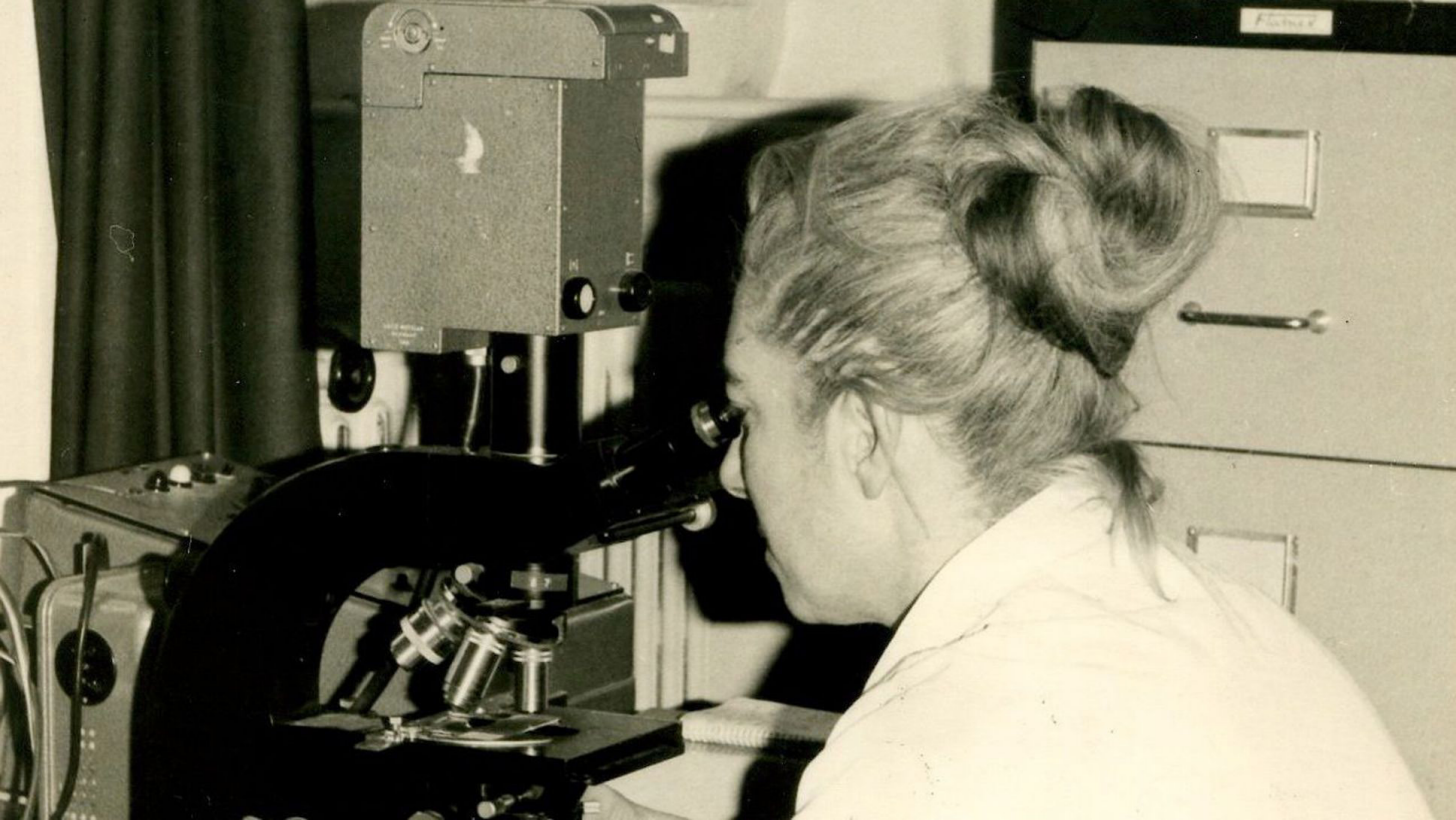
Marthe Gautier. Born in Montenils, France on 10 September 1925, Marthe Gautier trained as a paediatrician, and began research into Down syndrome in 1956. With no funding or facilities, she built a cytogenetics lab from scratch at the Hôpital Trousseau in Paris, and developed the techniques that later enabled her to reveal the presence of 47 chromosomes in children with Down syndrome. However, most of the credit for this work went to Jérôme Lejeune, one of her co-authors on the paper reporting this discovery. Marthe Gautier finally received recognition for her discovery in 2014. She died in 2022.

Marthe Gautier (Figure 4) is best known for discovering that individuals with Down syndrome have an extra copy of chromosome 21 (trisomy 21). Trained as a paediatrician under the mentorship of Robert Debré, one of the fathers of modern paediatrics, she was one of only two women in a class of 80 students to pass the internship exam for the hospitals of Paris. Later, in 1955, she was one of the first French students to earn a scholarship to go to Harvard University (Gautier, 2009), where she studied paediatric cardiology during the day, and she spent her weekends in a cell culture laboratory, where she learnt the techniques that would later serve her well in her research on Down syndrome.

Coming back to Paris in 1956 to find that the position she had been promised had been given to a man in her absence, Gautier transferred to Paediatric Unit at Hôpital Trousseau. The director of the unit, Raymond Turpin, had long been interested in Down syndrome, and was keen to investigate the implications of recent work that had established that healthy humans have 46 chromosomes. As no cytogenetic laboratories existed in Paris, Gautier established one from scratch to study the chromosomes of children with Down syndrome. With no institutional funding, she had to improvise, even using her own blood as a serum source (and also using her own savings to buy equipment). She developed and refined techniques that produced exceptionally clear chromosome preparations. Moreover, by training her own technicians and maintaining rigorous standards despite scarce resources, Gautier demonstrated that scientific ingenuity, determination and commitment could overcome even the most severe constraints.

When Gautier analyzed tissue from healthy subjects, she could clearly see 46 chromosomes. And when she analyzed tissue from children with Down syndrome, she could see 47 chromosomes. However, she did not have a light microscope, so she was unable to photograph her slides and document her discovery. Therefore, when Jérôme Lejeune, a researcher who was also working for Turpin, offered to photograph the slides for her, she agreed. There are competing versions of what happens next. Gautier says that Lejeune presented the work as his own in seminars, and that she – rather than Lejeune – should have been the first author on the paper reporting the results in 1959. To add insult to injury, her name was misspelt as Gauthier on this paper (Lejeune et al., 1959). Although Gautier, Lejeune and Turpin published further papers on Down syndrome together, for many years most of the credit for the discovery went to Lejeune, who was appointed to the first chair in human genetics at the Paris School of Medicine in 1964 and went on to receive a number of prizes for his work.

Lejeune died in 1994, but the Jérôme Lejeune Foundation continues to defend his name and contest Gautier’s claims. Indeed, in 2014, when the French Federation of Human Genetics awarded Gautier its grand prize, the lecture she had been invited to give at the federation’s biennial congress in Bordeaux was cancelled at the last minute because the organisers were worried that the Lejeune Foundation might take legal action (Casassus, 2014; Pain, 2014). However, later in 2014, Gautier’s crucial role in the discovery was formally recognized in a report by the Ethics Committee of Inserm, the agency that funds health and medical research in France (Inserm, 2014). According to this report: “The initial communication to the Academy of Sciences incorrectly listed Marthe Gautier’s name second.” Moreover, the report also stated: “Since the discovery of trisomy could not have been made without the essential contributions of Raymond Turpin and Marthe Gautier, it is regrettable that their names have not been systematically associated with this discovery, both in public statements and in the awarding of various honors.”

Marthe Gautier died in 2022, having had a long and successful career in paediatrics, and having finally reclaimed ownership of her discovery. Unfortunately, many women scientists cannot say the same.

On a positive note, Marthe’s name, along with the names of 71 other female scientists, will soon be engraved on the first floor of the Eiffel Tower, alongside the names of the 72 male scientists that have been present since the tower was opened in 1889.

## Making science more inclusive

For the development of science, it is important to organise scientific research in an inclusive way. We have to welcome and nurture talent, in all its diversity. We need to increase the recruitment of research staff from minority backgrounds, and we need to do more to retain staff so as to increase the diversity of those in leadership positions. This requires action at all levels, from institutions to the national and international levels. Particular attention should be paid to conferences, workshops and training courses by, for example, avoiding all-male panels (or “manels”) and providing onsite childcare (Else, 2019; Sardelis et al., 2017). Representation matters: our view of what a successful scientist looks like is influenced by what we see and what we hear and read about. We need to share, value and acknowledge the work of all scientists, regardless of their gender, nationality, ethnicity, sexual orientation or position within the laboratory. Science is richer and better when we include everybody.

Part of this involves recognizing those who have been overlooked despite making major contributions to science in the past, which is why we have written this article. Ethel Browne Harvey, Hilde Pröscholdt Mangold, Ida Henrietta Hyde, and Marthe Gautier – and many other women scientists – all deserve to be better known than they are.

## Data Availability

No data are associated with this article.
